# A comprehensive review of HIV/STI prevention and sexual and reproductive health services among sex Workers in Conflict-Affected Settings: call for an evidence- and rights-based approach in the humanitarian response

**DOI:** 10.1186/s13031-017-0124-y

**Published:** 2017-12-04

**Authors:** Alyssa Ferguson, Kate Shannon, Jennifer Butler, Shira M. Goldenberg

**Affiliations:** 1 Gender and Sexual Health Initiative, British Columbia Centre for Excellence in HIV/AIDS, St. Paul’s Hospital, 608-1081 Burrard Street, Vancouver, BC V6Z 1Y6 Canada; 2 Department of Medicine, University of British Columbia, St. Paul’s Hospital, 608-1081 Burrard Street, Vancouver, BC V6Z 1Y6 Canada; 3 United Nations Population Fund (UNFPA), Eastern Europe and Central Asia Region (EECAR), Istanbul, Turkey; 4 Faculty of Health Sciences, Simon Fraser University, Blusson Hall, 8888 University Drive, Burnaby, V5A 1S6 Canada; 5 Faculty of Health Sciences, Simon Fraser University, Gender and Sexual Health Initiative, BC Centre for Excellence in HIV/AIDS, St. Paul’s Hospital, 608-1081 Burrard St, Vancouver, BC V6Z 1Y6 Canada

**Keywords:** Sex work, Conflict, Post-conflict, Sexual and reproductive health, HIV/Aids, STIs

## Abstract

**Background:**

While the conditions in emergency humanitarian and conflict-affected settings often result in significant sex work economies, there is limited information on the social and structural conditions of sex work in these settings, and the impacts on HIV/STI prevention and access to sexual and reproductive health (SRH) services for sex workers. Our objective was to comprehensively review existing evidence on HIV/STI prevention and access to SRH services for sex workers in conflict-affected settings globally.

**Methods:**

We conducted a comprehensive review of all peer review (both epidemiological and qualitative) and grey literature published in the last 15 years (2000–2015), focusing on 1) HIV/STI vulnerability or prevention, and/or 2) access to SRH services for sex workers in conflict-affected settings. Five databases were searched, using combinations of sex work, conflict/mobility, HIV/STI, and SRH service terms. Relevant peer-reviewed and grey literature were also hand-searched, and key papers were cross-referenced for additional material.

**Results:**

Five hundred fifty one records were screened and 416 records reviewed. Of 33 records describing HIV/STI prevention and/or access to SRH services among sex workers in conflict-affected settings, 24 were from sub-Saharan Africa; 18 studies described the results of primary research (13 quantitative, 3 qualitative, 2 mixed-methods) and 15 were non-primary research (e.g., commentaries, policy reports, programmatic manuals). Available evidence indicated that within conflict-affected settings, SWs’ capacity to engage in HIV/STI prevention and access SRH services is severely undermined by social and structural determinants including widespread violence and human rights violations, the collapse of livelihoods and traditional social structures, high levels of displacement, and difficulties accessing already scant health services due to stigma, discrimination and criminalization.

**Discussion/Conclusions:**

This review identified significant gaps in HIV/STI and SRH research, policy, and programming for conflict-affected sex workers, highlighting a critical gap in the humanitarian response. Sex worker-informed policies and interventions to promote HIV/STI prevention and access to HIV and SRH services using a rights-based approach are recommended, and further research on the degree to which conflict-affected sex workers are accessing HIV/STI and SRH services is recommended.

A paradigm shift from the behavioural and biomedical approach to a human rights-based approach to HIV/STI prevention and SRH is strongly recommended.

## Background

Forty armed conflicts were active in 2014, an 18% increase when compared to the 34 reported in 2013, with many additional countries currently considered fragile states, or involved in post-conflict rehabilitation [1]. Armed conflicts have resulted in unprecedented waves of population displacement as well as other deleterious human rights, public health, and social impacts, including the disruption of traditional social structures, a breakdown in security, and weakened or collapsed health systems [2–5].

Roughly 50% of the estimated 43 million people made refugees or displaced by conflict are women [6]. While the relationship between conflict and HIV/STI prevalence in the general population is greatly shaped by contextual factors [7, 8], and has been found to vary by setting, women have been shown to often be disproportionately vulnerable to the negative health and social consequences of displacement [9, 10]. The economic, social and political instability of conflict and post-conflict environments, including social and physical displacement, loss of traditional economic options, cultural upheaval, family separation and increased women-headed households, often result in conditions that facilitate significant engagement in sex work as a source of income, particularly for women. Further, armed conflict and the highly policed and militarized environment characteristic of post conflict have been linked to widespread gender-based violence (including rape as a weapon of war; forced abductions), rights violations of women [3, 11–17], and reduced access to, or the interruption of, HIV and sexual and reproductive health (SRH) programmes [4, 18–21]. For example, previous research has shown that within conflict settings, the interruption of condom distribution, disruption of HIV diagnostic services, and shortages of HIV antiretroviral therapy (ART) may drastically impede diagnosis and care [18]. Together, these dimensions of conflict create a complex and challenging situation for prevention of HIV/STIs and delivery of care to conflict-affected populations [10], yet the lived experiences of sex workers, conditions within post-conflict environments, and barriers to accessing HIV and SRH services have largely been unaddressed in research and policy. Furthermore, that programmers and development partners in humanitarian settings may be uninterested in sex work or may conflate issues of sexual exploitation with sex work for ideological or political reasons, creates perverse barriers in the protection of human rights for this group.

Sex workers are a key population disproportionately affected by HIV/STIs [22]. While the majority of sex workers globally are women, there are sizable populations of men and transgender sex workers in many settings [23–25]. HIV/STI prevalence among sex workers varies both across and within regions due to structural factors related to the social, political, economic, legal, and cultural conditions in which sex workers operate, in conjunction with local HIV and STI epidemics [9, 22, 26]. Despite this, research and programmes in the past decade have largely focused on behavioural and biomedical interventions among SWs, which alone, have had only modest effects on the reduction of HIV at the population-level [22, 27]. A recent global review identified a critical need for further studies examining structural HIV/STI risks or access to care for sex workers in the highest-HIV burden countries [9], to inform the design, adaptation and implementation of effective HIV/STI programmes, particularly needed within conflict-affected settings of sub-Saharan Africa. While sex workers are often highly marginalized even in non-conflict settings, in conflict-affected environments they may face elevated social and structural risks and barriers to care, including abuses of human rights by military and police, gender-based inequities, widespread violence, discrimination and stigma, social and physical isolation, breakdowns in health service delivery systems, and other structural risks that often accompany or follow a crisis [11, 14, 26, 28, 29]. Despite this, little is known about conflict-affected sex workers’ vulnerability to HIV/STIs or access to HIV and SRH services, or their social and structural drivers within conflict-affected settings. Given the paucity of existing data regarding HIV/STI risks and access to HIV and SRH services within the context of sex work in conflict-affected settings, this comprehensive review aimed to broadly explore and synthesize current evidence on HIV/STI risk, access to HIV and SRH services, and their social and structural determinants within the context of sex work in conflict-affected settings (i.e., conflict and post-conflict conditions).

## Methods

### Search strategy

**Fig. 1:**
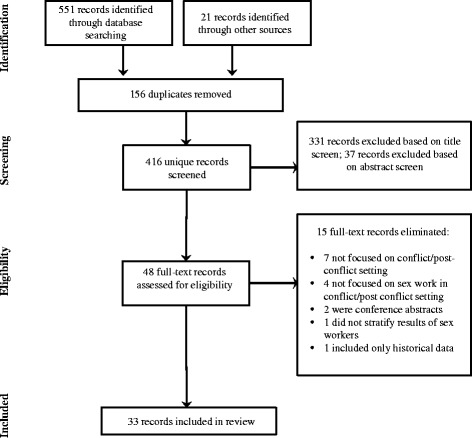
PRISMA

From May to July 2015, we comprehensively searched the peer-reviewed and grey literature for material describing HIV/STI risk or prevention and/or access to HIV or SRH services for sex workers in conflict-affected settings in the last ten years. Five databases (PubMed, Global Health, PAIS International, Social Sciences Citation Index, and Web of Science Core Collection) were searched using combinations of terms related to sex work, conflict, HIV/STI risk or prevention, and HIV and SRH services access related terms (Table 1). Relevant journals and organizational websites were hand-searched, and key papers were cross-referenced. Due to the limited number of relevant peer-reviewed studies available, grey literature (e.g., governmental and non-governmental reports) was searched. Studies conducted with populations of relevance other than sex workers (e.g., Internally Displaced Persons (IDPs), clients of sex workers) were also considered and included where they provided useful context and insight regarding sex work and HIV, STI, or SRH issues in conflict-affected settings. The first and second rounds of screening involved reviewing titles and abstracts, respectively, to identify potentially relevant studies. The third-level of screening consisted of a full-review of remaining records to ascertain relevance in relation to the inclusion criteria. We used the PRISMA guidelines as a reference (Fig. 1).

**Table 1:** Search terms

Sex work	“sex work*” OR “prostitute*” OR “transactional sex” OR “commercial sex” OR “sex trade” or “FSW*”
Conflict environment	“conflict” OR “emergenc*” OR “IDP” OR “displaced” OR “displaced person*” OR “displaced people” OR “refugee*” OR “humanitarian” OR “war”
HIV/STI risk or prevention, and HIV and SRH services	“HIV” OR “human immunodeficiency virus” OR “HIV infections” OR “AIDS” OR “acquired immunodeficiency syndrome” OR “acquired immune deficiency syndrome” OR “sexually transmitted infections” OR “STIs” OR “Sexual health services” OR “sexual health” OR “reproductive health” OR “testing” OR “test” OR “treatment” OR “ART” OR “ARVs” OR “sex education” OR “sexual health education” OR “safer sex” OR “contraceptives” OR “birth control” OR “family planning” OR “pap smear” OR “condoms” OR “health services” OR “health care” or “healthcare”

### Inclusion criteria

Eligible records included peer-review publications (qualitative, quantitative, or review articles) or grey literature (e.g., policy documents, community reports, commentaries, issue reports and briefs, position reports, practical guides for staff working in conflict/post-conflict environments) that met the following criteria: 1) English literature; 2) published from January 2000–July 2015; 3) discussed sex workers (or their clients) working within conflict or post-conflict settings (e.g., refugee or internally displaced sex workers); and 4) included data on HIV/STI risk, prevention, or HIV and SRH services for sex workers in conflict-affected settings. Other reviews were included, as they synthesized key insights in a domain with a paucity of empirical studies. For the purpose of this review, the United Nations definition of sex work was adopted, defined as the sale or exchange of sex for accommodation, protection, food, gifts and other items or services. Studies which were explicitly and solely focused on transactional sex (i.e., broader populations of those who exchange sex for favours or gifts, or who do not necessarily self-identify as sex workers) were excluded. Studies of the trafficking in human beings for the purposes of sexual exploitation were also excluded, unless they included the experiences of sex workers. Conflict-affected sex workers included those who identified as refugees or IDPs from conflict or post-conflict settings (defined as ≤10 years post-conflict) as well as sex workers currently operating in conflict-affected (i.e., current or post-conflict) settings.

### Data extraction and analysis

Endnote was used to manage retrieved items. A Microsoft Excel database was developed to organize and chart study characteristics (authors, year, country, design, population, sample size), key findings, and the following data, where applicable: HIV/STI prevalence, conflict-related variables, qualitative findings, and key programme and policy recommendations. We began by grouping the findings of the epidemiologic studies according to common topics and structural determinants, comparing them across studies. Next, we elicited common themes from the qualitative data and compared these across settings. Lastly, we analyzed existing refugee and sex worker HIV/STI prevention and SRH programme and policy recommendations in conflict settings, seeking to draw lessons and exemplars for future programmes and interventions.

## Results

Five hundred and fifty-one titles and abstracts were screened by the first author to determine eligibility. Four hundred and sixteen eligible records were reviewed. Of the 33 records which met the criteria to be included in this review, the majority (*n* = 22) described conflict or post-conflict environments in sub-Saharan Africa, 4 were from other settings (e.g., Afghanistan, Bangladesh/Myanmar, Sri Lanka, and Nepal), and 7 were globally focused. Eighteen studies described the results of peer-reviewed primary research articles (13 quantitative, 3 qualitative, 2 mixed methods) (Table 2), and 15 were non-empirical research (e.g., review articles, commentaries, issue reports and briefs, position reports, and practical guides for staff working in conflict/post-conflict environments). A summary of practical guides for staff working in conflict/post-conflict environments (*n* = 5) are described in Table 3. Twenty four of the 33 records were peer-reviewed, while 9 were classified as grey literature/non-peer reviewed. Of the 33 included studies, 14 focused primarily on sex workers in conflict/post-conflict settings, while others discussed broader dynamics of sex work (e.g., sex purchasing) among the general conflict-affected population (*n* = 17), sex workers’ clients (e.g., migrants, combatants) (*n* = 5), or a combination of these groups. While eligibility was inclusive of all gender and sexual orientations, the majority of studies reported on cis-gender female sex workers. One study reported on sexual and gender minority sex workers in displacement and post-conflict settings [23].

**Table 2:** Characteristics of primary studies pertaining to sex work and HIV, STIs, and SRH in conflict-affected settings

Reference	Location	Design	Population (N)	Stage of conflict studied	Key Findings
Quantitative (13)					
Alemayehu et al. (2015) [32]	Mekelle City, Ethiopia	Cross-sectional	SWs (*N* = 250)	Post-conflict	*STI history:* 17% reported history of an STI, gonorrhea (45.8%) syphilis (41.7%), and chancroid (12.5%) *STI testing:* 9.6% of those with an STI reported having sought treatment *Reproductive health*: 27% of SWs reported a history of at least one elective abortion, with 35.3% of these women reporting more than one pregnancy termination. *Contraceptive use*: 69% of SWs acknowledged any type of contraceptive use *Violence:* Prevalence of sexual violence among SWs = 75.6%, correlates included lower education, sex work duration, and drug use. SWs with lower monthly income were the most likely to experience sexual violence.
Bing et al. (2008) [50]	Angola	Cross-sectional (Behavioural surveillance study)	Military personnel (*N* = 1710)	Post-conflict	*Combatants as sex buyers:* 9% of combatants reported having sex with a SW in past 12 months *Condom use:* 54.2% of military personnel reported using a condom at last sex with a SW *STIs:* Combatants who had casual sex partners or who had sex with a SW during the past year were significantly more likely to report STI symptoms than those without such sexual partners.
Dupas et al. (2012) [43]	Kenya	Retrospective study	- SWs (*N* = 248) - Self-employed entrepreneurs (*N* = 230) - Shopkeepers (*N* = 325)	Active and post-conflict comparison	*Influence of political violence on unprotected sex:* SWs engaged in higher risk (unprotected vaginal or anal) sex both during and after the post-election crisis, to make up for income shortfall. Overall levels of higher risk sex declined during the crisis, but women responded to the negative income shock by significantly increasing the amount of unprotected sex they had, conditional on being able to find clients.
Erickson et al. (2015) [31]	Gulu, Uganda	Cross-sectional	SWs (N = 400)	Post-conflict	*HIV/STI prevalence:* 22.3% SWs reported HIV infection and 40.3% reported STIs *Contraceptive use:* 45.0% of SWs used male condoms and non-barrier family planning methods. *Policing:* Having to rush sexual negotiations owing to police presence was negatively associated with dual contraceptive use (AOR 0.65, 95% CI 0.42–1.00; *P* = 0.050). *HIV testing:* Dual contraceptive use was positively associated with HIV testing (AOR 5.22, 95% CI 1.75–15.57; *P* = 0.003), suggesting the potential importance of better integration of HIV/SRH services.
Goldenberg et al. (2015) [11]	Gulu, Uganda	Cross-sectional	SWs (*N* = 400)	Post-conflict	*HIV infection:* 33.75% of SWs were HIV-seropositive (compared to 8.51% of women of reproductive age in general pop); of whom 33.3% were new/previously undiagnosed HIV infections. *Abduction by rebels:* War-related abduction was associated w/HIV (AOR: 1.62, 95% CI: 1.00–2.63). *Criminalization:* Incarceration (AOR: 1.93, 95% CI: 1.17–3.20) associated w/ HIV
Harrison et al. (2009) [45]	Oruchinga and Nakivale refugee settlements, Uganda	Cross- sectional (Standardised behavioural surveillance survey (BSS))	- Settlement refugees who sold sex in last 12 months (*N* = 93) - Ugandans in surrounding settlement area who sold sex in last 12 months (*N* = 47)	Post-conflict	*Sex work following displacement:* More refugees than nationals reported exchanging sex for money, drugs or other goods (10% versus 6%; *p* < 0.01), which mostly occurred post-displacement. Sex work engagement higher in the refugee population vs. Ugandan nationals (4.7% vs. 2%). *Condom use:* Condom use was low in both populations, but lower among refugees. Condom use at last sex with all types of partners (non-regular, paid, and higher risk) four-times higher among the nationals than refugees, but confidence intervals overlapped. *Sexual violence:* Percentage of women aged 15–59 forced to have sex in the past year was roughly the same for refugees (1.3%) as nationals (1.2%).
Kriitmaa et al. (2010) [54]	Hargeisa, Somaliland, Somalia	Cross sectional (Integrated bio-behaviouralsurveillance (IBBS))	SWs (*N* = 237)	Post-conflict	*HIV infection:* Heterosexual commercial sex suggested as dominant mode of HIV transmission *Condom use and access:* 24.0% SWs reported using a condom at last SW transaction and only 4.3% reported consistent condom use with clients in the past month. Of the 24.0% who did use a condom at last sex with a client, 80.5% said it was suggested by the client. 29.5% didn’t use condoms with clients due to not knowing where to obtain condoms. Almost none (0.4%) received condoms through a clinic or outreach in the past year. *HIV testing:* Only 2.6% SWs knew where to go for a confidential HIV test. 4% reported ever having had an HIV test, and none of them received their test results. *HIV prevention:* 6.9% SWs correctly answered all 5 questions on HIV factual knowledge; only 38.4% had ever heard of an STI.
Larsen et al. (2004) [36]	Sierra Leone	Pre-post test intervention	- SWs (*N* = 202) - Military (*N* = 205)	Post-conflict	*HIV knowledge:* Only 8.5% SWs and 22.8% military knew >3 modes of HIV transmission *Condom knowledge/access*: 14.9% of SWs and 12.4% of military knew no sources to purchase condoms
International Office of Migration (2008) [35]	Hargeisa, Somalia	Cross-sectional (IBBS)	SWs (*N* = 219)	Active conflict	*HIV knowledge*: No SWs knew their HIV status, 93% SWs lacked correct HIV prevention knowledge. *Condom use:* 28% SWs had never used a male condom *Migration:* 69% SWs were migrants
Muldoon et al. (2015) [14]	Gulu, Uganda	Cross-sectional	SWs (*N* = 400)	Post-conflict	*HIV seroprevalance:* 33.8% *SW demographics:* Sample was generally young, the majority between the ages of 19–25 yrs., many with dependent children. 65% of SWs had less than primary school education *Violence:* 49.0% of SWs reported extreme physical and/or sexual workplace violence in the previous six months, including physical assault, rape, and gang rape. Among 196 SWs who reported client violence, the most common forms included being physically assaulted (58.7%), raped (38.3%), the client attempting sexual assault (18.4%), and being gang raped (15.8%). *Condom use:* 84.0% SWs reported inconsistent condom use with regular or one-time clients. *Policing*: Rushing negotiations due to police presence contributed to client violence (AOR: 1.61, 95% CI: 1.03–2.52). Highlights negative consequences of policing practices for conflict-affected SWs.
Ntumbanzondo et al. (2007) [44]	Kinshasa, Democratic Republic of the Congo (DRC)	Cross-sectional	SWs (*N* = 136)	Active conflict	*Sexual decision making*: 96.3% SWs felt they were able to negotiate safer sex with clients. *Condom use*: 81.6% SWs always used a condom with clients, but 26.5% reported charging extra for unprotected sex with clients upon request. *Unprotected sex for more money:* SWs who engaged in unprotected sex for more money were significantly more likely to live or work at non-downtown sites (OR = 3.07), and to have at least one child less than six years of age (OR = 2.95). They charged a median of 2.90 USD (IQR: 1.54 USD 4.61 USD) for protected intercourse. The median ratio of their charge for unprotected intercourse to their charge for protected intercourse was 3.5 (IQR: 2.5 5.0). *HIV Knowledge*: ~75% of SWs feared contracting HIV as a result of unprotected intercourse.
Rowley et al. (2008) [49]	Tanzania	Cross-sectional	Refugees/IDPs aged 15–24 exchanging sex (*N* = 16) Surrounding villagers aged 15–24 exchanging sex (*N* = 32)	Post-conflict	*SW engagement among refugees:* 40% of refugee/IDPs 15-25 yrs. reported exchanging sex for money, gifts, or favors during the last year, compared with 21% of village respondents (χ2 33.83, *p* = .000). *Condom use*: Condom use at last sex with non-regular or paid/transactional partners amongst refugees/IDPs (40%), compared to village respondents (21%).
Todd et al. (2011) [53]	Afghanistan	Cross-sectional	SWs (*N* = 520)	Active conflict	*SW demographics*: 76.9% SWs had no formal education *Displacement/mobility*: 37.7% SWs lived outside Afghanistan in the last five years. *HIV knowledge:* Only 17.4% SWs had comprehensive HIV knowledge. *Condom knowledge*: <60% SWs heard of condoms; of those who had, only half had used a condom.*Condom use*: Consistent client condom use was reported by 11.5% SWs and was independently associated with having more clients per month.
Qualitative (3)					
Maclin et al. (2015) [37]	Goma, Bukavu and Kalehe, DRC	Focus group discussions	- Vulnerable women - Males involved in community groups	Post-conflict	*Post-conflict SW seen to break down traditional social structures/family dynamics*: Discussants detailed how exchanging sex (i.e., commercial or transactional sex) post-conflict was linked to poverty. This was seen to undermine traditional social structures and family dynamics, and was portrayed as both a symptom of, and a catalyst for, changes within family dynamics resulting from conflict-related experiences in eastern DRC. Families were physically separated because of the conflict – from death, displacement and marriage dissolution, according to study participants.
Muhwezi et al. (2010) [38]	Katakwi and Amuria, Uganda	Focus group discussions Key informant interviews In-depth case study interviews	- FGD (4 men, 4 women) - KI (16 men, 16 women) - Case studies (8 men, 8 women)	Post-conflict	*Sex work as a consequence of conflict:* Breakdown of the social structure due to conflict resulted in economic destruction and a perceived soaring of vulnerable people whose propensity to engage in high-risk sexual behaviour was increased. *High risk sexual behavior due to refugee camp environment*: High risk sexual behaviour (lifestyle or activity that places a person at increased risk of suffering or being infected with HIV/AIDS, a sexually transmitted disease and/or an unwanted pregnancy) was associated with concentration of people in camps where idleness and unemployment were the norm.
Nyanzi (2013) [23]	Kampala, Uganda	Repeat individualin-depth interviews Repeat focus-group discussions	- 54 wacheche (SWs and same-sex-loving or gender non-conforming people)	Post-conflict	*Criminalization perpetuates violence against SWs:* Perpetrators of ‘hate-crimes’ against SWs, went unpunished numerous times because the victim refused to report the case. *Criminalization creates barriers in access to services:* In criminalized states where SWs are policed, individuals may fail to access available services for fear of disclosing their so-called ‘non-conforming sexual practices’. *Displacement impedes legal knowledge:* Displaced persons in Uganda found to have varying levels of awareness of the legal frameworks governing sexual conduct and relationships within their contexts of displacement.
Mixed-Methods (2)					
Gazi et al. (2008) [47]	Teknaf, Bangladesh/ Myanmar	Cross-sectional survey & KI interviews	Boatmen (*N* = 433)	Active conflict	*Migrants as sex buyers*: 17% of Bangladeshi migrant boatmen reported having had sex with a SW while in Myanmar. *Prolonged displacement and sex buying:* Significant correlation found between the number of nights spent away from home and engaging in paid sex. *Condom use:* Condom use by migrants with SWs and other partners was rare (0–4.7% in the last month), and did not vary with types of sex partners.
International Office of Migration (2012) [48]	Somalia	HIV Hotspot Mapping	- SWs (*N* = 143) - Male clients (*N* = 73)		*Violence:* SWs commonly reported assault, threats, violations, ill treatment, rape, and refusal to pay by clients, resulting in injury and damage to property, reduced ability to work, and loss of income. *Mobility*: SWs reported high levels of mobility before and after engagement in paid sex. Many left home after losing parents/caregivers, or experiencing conflict and domestic violence. Transactional sex more prominent due to population movements: SWs in Somaliland and South-central more likely to report exchanging sex for gifts or favors rather than money. *SW entry linked to economic survival:* SW initiation typically referred to as a means of survival during conflict. *HIV testing:* Most SWs had never been tested for HIV and did not know their status; main barrier to testing was a lack of risk perception. Many doubted the confidentiality of VCT services. Those who sought to test were motivated by illness among a fellow SW. *HIV knowledge:* Almost all migrant SWs had heard of HIV, but knowledge around prevention and transmission was mixed, with many misconceptions still present. *Migrants as sex buyers:* Most common sex clients were truck drivers, seafarers, port workers, uniformed services, businessmen, traders and unemployed men.

**Table 3:** Summary of evidence on HIV/STI and SRH policies and programmes for conflict-affected sex workers

Reference	Document title	Population	Key programmes, policies and/or recommendations
Inter-Agency Working Group (IAWG) on Reproductive Health in Crises [55]	Inter-agency Field Manual on Reproductive Health in Humanitarian Settings	Refugee/IDPs	*Sex worker sensitive HIV VCT programmes:* Behaviours that put people at a higher risk of exposure to HIV, such as sex work or injecting drug use, also make people more susceptible to coercion, discrimination, violence, abandonment, incarceration or other negative consequences upon disclosure of an HIV-positive test. Healthcare providers require special training and supervision to uphold standards of informed consent and confidentiality for these populations. HIV VCT for these groups should be accompanied by the implementation of a supportive social, policy and legal framework. *Condom availability/distribution:* Consult with local staff about how condoms can be made available in a culturally sensitive way, particularly for most at-risk groups, such as SWs and their clients, MSM, IDUs and young people. Ensure the consistent availability of quality male and female condoms. To see an effective reduction of HIV transmission through SW requires >90% compliance of correct use of condoms among SWs and their non-regular sex partners. *Spermicides:* Not recommended for SWs, as they increase risk of HIV *SW specific services:* Recommended at health service level, RH officers recommended to hot-spot areas where SWs congregate to target interventions and services *STI screening:* Service providers recommended to offer regular screening to people with frequent exposure to STIs, such as SWs *STI treatment*: Presumptive treatment of SWs recommended at first visit followed by regular visits for speculum/bimanual examination and Gram stain of cervical smear. *Right to equality and non-discrimination:* Protected by providing access to STI services for the entire population, including adolescents, SWs and MSM, regardless of the legal status of prostitution and homosexuality in a country *Inclusion of SWs in programming:* Involve vulnerable groups encouraged to be involved from the start in programme design, implementation and monitoring. *Violence reduction strategies:* Should be integrated in SW settings. Programmes recommended working with law enforcement to ensure SW’s ability to protect themselves and to ensure safer sex practices by their clients. *SW and child protection:* Communities and SWs should be engaged in child protection policies and regulations. *Offer exit-strategies*: Programmers encouraged to link SWs and their families to support mechanisms, including the provision of assistance and incentives for women to leave sex work through a range of legal, economic and social services. *Address sex buyers:* Work to change the behaviour of SWs’ clients (humanitarian staff, peacekeepers, police, general population) *HIV prevention for vulnerable groups:* Involve groups from the start in programme design, implementation and monitoring; locate programme activities in places frequented by the group (clubs, neighbourhoods, etc.); create safe virtual (telephone hotlines) or physical (drop-in centres) spaces tailored to the group; train health and social workers to provide high-quality, client-friendly, HIV-related services; address structural barriers, including policies, legislation and customary practices, that discriminate against the group and prevent access and utilization of appropriate HIV prevention, treatment and care services.
UNAIDS Inter-Agency Task Team on Gender and HIV/AIDS (2001) [16]	HIV/AIDS, Gender and Conflict Situations	Refugee/IDPs	*HIV/STI programmes:* National governments, national and international NGOs and UN agencies encouraged to incorporate STI and HIV prevention measures into all humanitarian assistance. Donors strongly encouraged to support these interventions. *Conflict, displacement, HIV and gender inequality research:* Assessments encouraged to be carried out, in collaboration between government and agencies, to determine the links these factors in each humanitarian situation. Steps encouraged to be taken to ensure that all humanitarian programmes are responsive to issues documented in these assessments. *Focus on women:* All HIV/AIDS programmes and funding in conflict situations encouraged to address the disproportionate disease burden carried by women. Effective approaches include sensitisation, training and behaviour change communication programmes targeting men and boys as well as women and girls. *International guidelines for peacekeepers:* Steps encouraged to be taken to ensure the implementation of internationally agreed guidelines for the prevention of HIV transmission during peacekeeping operations. Peacekeepers encouraged to receive training on women’s rights and gender-based violence as well as HIV prevention. Because peacekeepers have sometimes been implicated in abuses against women and girls, mechanisms of accountability encouraged to also be included. *Sexual violence programming:* Programmes encouraged to be designed to support the victims of sexual violence through medical care, counselling, support groups and related activities. Health service packages for girls and women who have been raped encouraged to include post-exposure HIV prophylaxis. *Military HIV/STI programming:* Programmes encouraged to be undertaken to improve HIV/STI awareness and treatment within the regular military and rebel forces, where these are systematically demobilised. This will have important impacts on sexual health risks to civilians from ex-combatants. Civilians, including SWs near military installations, encouraged be included in these awareness raising and treatment programmes.
UNHCR (2010) [67]	HIV and sex work in refugee situations: A practical guide to launching interventions an issue affecting women, men, girls, boys and communities	Conflict-affected SWs	This guide is intended to assist those working to slow transmission of HIV and other STIs in humanitarian settings. The focus is on intervening where HIV has the potential to spread quickly – with SWs and their clients. Practical, step-by step activities are recommended for addressing HIV in sex work within refugee situations, including: *1. Sensitization and buy-in:* Engage agencies responsible for refugees, community groups, and leaders. *2. Identification, hotspot mapping and snowballing:* Collect baseline and risk information, provide condoms, and assess need through snowball sampling, mapping hotpots, and estimating numbers of SWs and clients. *3. Protection:* Support registration, ensure safe access to basic needs, and reinforce GBV prevention and child protection activities *4. Profiling and case management:* Gain deeper understanding of sex work in the community, identify those most at vulnerable/risk, and develop case management plans to address urgent problems *5. Forming Multi-Functional teams (MFTs):* Guide programme implementation, identify roles and responsibilities*,* strengthen partnerships, and ensure coordination and monitor progress *6. Building peer-led systems with SWs:* Meet and review programme objectives, introduce verbal contract about participation in peer group, ask SWs to choose their leaders and agree to meet regularly, provide peer leaders with training, condom education, promotion and distribution kits *7. Health services:* Assess services looking at HIV/STI-related areas, advocate and ensure SWs have access to non-judgmental services *8. Male and venue-based interventions:* Engage men and boys, and offer simple venue-based interventions *9. Monitoring*
UNFPA & UNHCR – Burton et al. (2010) [3]	Addressing HIV and sex work	Conflict-affected SWs	Interventions to respond to HIV and sex work in humanitarian settings are both necessary and feasible, even during an emergency. In situations where comprehensive HIV programmes have already been established but where SWs have not yet been reached, a basic set of sustainable multisectoral activities can be established within six months. Key Activities per phase include: Preparedness *1. Integrate HIV and sex work into contingency planning*: Identify existing SW networks and programmes, map services, and develop contingency plans for rapid restoration if disrupted Emergency phase *2. Expedite registration, risk identification and protection*: Identify those most at risk, ensure protection, establish GBV services, and promote codes of conduct *3. Ensure safe shelter and access to food and basic necessities* *4. Provide basic SRH and HIV services:* Implement MISP*,* establish basic STI/SRH services and outpatient clinics, and implement basic HIV services *5. Start outreach:* Begin mapping and engagement with SWs, identify sex-work venues, and distribute condoms and information Stabilised phase *6. Build supportive environments and partnerships:* Establish peer groups, support SW-led approaches, strengthen existing women’s groups to reach non self-identified SWs, conduct rapid assessments, and plan interventions *7. Reinforce protection:* Strengthen prevention of GBV and sexual exploitation, and find ways to involve men *8. Expand to comprehensive HIV and SRH services including STI services* *9. Expand targeted services:* Support transition of peer activities to broader community mobilisation, strengthen venue-based and special clinics for identified SWs, and work with clients to reduce demand for unprotected paid sex *10. Provide social/economic/legal services:* Strengthen legal protections and establish self-regulatory boards, increase livelihood and educational opportunities for the most vulnerable, and prepare for appropriate durable solutions
Inter-Agency Task Team on HIV and Young People (n.d) [12]	HIV Interventions for Young People in Humanitarian Emergencies	Conflict-affected youth	*Provision of basic health care and support:* Providing basic health care and support to younger, most-at-risk groups, such as people who inject drugs (PWID), SWs and MSM. Special attention is needed to address the needs of younger age groups, particularly during the minimum response stage.

### Sex work context and links to HIV/STI prevention and risk in conflict-affected settings

Sex workers in conflict and post-conflict settings were found to face an extraordinarily high HIV and STI burden. The burden of HIV among sex workers in studies reviewed ranged considerably, with prevalence rates of 70% reported among Nepalese sex workers returning from India [30], and 22.3% among conflict-affected sex workers in Gulu, northern Uganda [31]. STI prevalence also ranged considerably, with an acute self-reported STI prevalence of 40.3% in Gulu, Uganda [31], and 17% of sex workers in northern Ethiopia self-reporting history of an STI [32]. Gonorrhea was the most common STI reported in this study, accounting for 45.8% of the total [32].

#### Gendered economic impacts of conflict and sex work entry

Available data indicated high rates of sex work in conflict and post-conflict settings globally [11, 30, 33]. A study in Nepal revealed that roughly 19.0% of sex workers reported having entered sex work directly because of subsistence needs attributed to local conflict [30]. Our review of both the qualitative and the quantitative literature pointed to the ways in which poverty, diminished employment opportunities, difficulty meeting subsistence needs, and challenges to sustainable livelihoods in contexts of displacement influenced engagement in sex work within conflict-affected settings [3, 5, 11, 17, 23, 30, 32–41].

Within the context of family separation resulting from armed conflict, increases in female-headed households were common, and women often experienced reduced access to traditional economic livelihoods, particularly in the absence of male support [36, 37]. Stemming from unequal gender norms and limited economic opportunities for women, existing evidence highlighted how women were particularly likely to exchange sex for money, accommodation, or other goods (e.g., food, clothing, healthcare, favours, gifts, access to education for their children, protection), and even passage across a border [3, 23, 33, 36, 41]. The following testimonial from a Somalian sex worker highlighted how the unequal gendered socio-economic impact of conflict influenced her choice to engage in sex work:

"[The first time I sold sex] I was 12 years old ... my father and mother had died … I didn’t have anyone to take care of me, and [didn’t] have anything to eat or dress in. Being a sex worker was the only choice." (Somalian sex worker aged 25 [35].

Inconsistent condom use within conflict-affected settings was commonly elucidated as a consequence of reduced negotiating power due to financial constraints during times of social unrest [30, 36, 39, 40]. Conflict-affected sex workers in sub-Saharan Africa commonly supported dependent children [14, 42], and having children (especially young children) was associated with increased HIV risks including engaging in unprotected sex for more money [14]. Although further research is needed in this area, two studies suggested increased need/opportunity to charge extra money for unprotected intercourse during or following a crisis [43, 44], including a study of Kenya’s 2008 post-election violence on sex workers and their clients, where dramatic declines in income, expenditures, and consumption in the general population resulted in more unprotected sex [43]. These findings elucidate potentially important effects of active vs. post-conflict settings on the sex industry, where conflict environments with a large presence of military/peacekeeping personnel as potential clients may increase demand for sexual services, (while reducing sex workers’ control to safely negotiate condom use with clients), whereas smaller social and economic crises (represented by dramatic declines in income) may result in more sex workers willingly offering unprotected sex to make up for income shortfalls. Authors further highlight the increased risk for sex workers during times of political and economic crisis, where financial gains of unprotected sex may outweigh the known risk of HIV/STI acquisition. Although sex workers in non-conflict settings also often report economic pressures for unprotected sex for higher pay, our review suggested that these pressures may be amplified within conflict-affected settings.

#### Displacement and mobility

Displacement and high mobility were common experiences reported by conflict-affected sex workers [11, 35, 45, 46], and clients of sex workers [35, 41, 45, 47], across diverse geographical and socio-political contexts. Within the studies reviewed, displacement and mobility were consistently associated with increased engagement in sex work [35, 41, 45, 48, 49], particularly in the post-conflict stage [35, 45, 48, 49]. While military/peacekeepers commonly purchase sex in conflict-affected environments [2, 16, 34, 38, 41] displacement may increase demand for sex work from other clients particularly during the rehabilitation stages of a conflict, due to men travelling greater distances for employment, and spending greater amounts of time separated from family [37]. Further, in sub-Saharan Africa, the geographical areas most heavily affected by HIV are often those linked with high long-term mobility, adjacent to main transport routes or in border regions [46].

Findings from numerous contexts, predominantly within sub-Saharan Africa, indicated that women in transit or temporarily displaced due to war or violence were more likely to report having received money or goods for sex than their non-mobile counterparts [26], and this was often linked to enhanced challenges and barriers accessing HIV and SRH services. In a study seeking to understand the relationship between armed conflict and HIV infection among conflict-affected sex workers in Gulu, northern Uganda, 66.5% of respondents reported living in IDP camps, among whom HIV prevalence was 71.9% compared to 63.8% of non-mobile sex workers [11]. In Somalia, mobility of sex workers was further associated with increased HIV/STI risk due to porous borders with countries that have higher HIV prevalences (Djibouti, Ethiopia, Kenya), and the presence of mobile populations associated with other HIV epidemics in the region (e.g., truck drivers and the military) [35].

#### Presence of military and peacekeepers

The presence of military and peacekeepers with expendable incomes represent unique aspects of conflict-affected settings which strongly shape the context of sex work and HIV/STI prevention in such contexts [2, 3, 5, 16, 29, 33, 34, 36, 38, 39, 41, 50]. Combatants and peacekeepers stationed far from home were more likely to frequent sex workers during war and migration [5], had higher rates of STIs than the general population [16], and were more likely to engage in higher-risk behaviour while participating in missions than within their home communities [2]. Even during peacetime, STI rates among armed forces are generally 2–5 times higher than in civilian populations; in times of conflict, the difference can be 50 times higher or more [39]. The circumstances of military service were said to make soldiers both more vulnerable to HIV/STI infection and more likely to transmit such infections – for example, soldiers are typically young, male, sexually active, and separated from their normal partners, which has been shown to facilitate sex purchasing [39]. Peacekeepers may also originate from countries with higher HIV prevalence rates than the local community in conflict, potentially exposing sex workers to further risk [36]. In Gulu, northern Uganda, exposure to war and conflict related events, such as abduction by the Ugandan Lord’s Resistance Army (LRA), was found to significantly increase the likelihood of HIV seroprevalence in sex workers (AOR: 1.62, 95% CI: 1.00–2.63) [11].

#### Violence

Physical, sexual, and emotional violence against sex workers in conflict-affected settings, by both clients and intimate partners was alarmingly high within studies [11, 14, 31, 32, 35, 37, 38]. Among sex workers in post-conflict northern Uganda, almost 50% experienced extreme physical and/or sexual workplace violence in the previous six months, including physical assault, rape, and gang rape [14]. Reports of assault, poor treatment, and threats were commonly reported by sex workers in Somalia, as were rape and refusal to pay [35]. An epidemiological study of 250 sex workers in post-conflict Mekelle, northern Ethiopia, demonstrated high rates of violence, with 46–60% of sex workers reporting rape or forced sex [32]. In Somalia, incidents of violence most often led to physical injuries and damage to property, reducing sex workers’ ability to work, creating medical costs, and leading to a loss of income [35], which in turn, may lead to a greater likelihood of engaging in sex for more money. In conflict-affected Gulu, northern Uganda, 74.7% of sex workers reported that they would experience violence from clients, and 60.8% from intimate partners, if they asked them to wear a condom [31]. Prolonged exposure to traumatic conflicts was found to be linked to severe mental distress, including persistent feelings of despair, hopelessness, pathological fear, and suicidal inclinations, which may have indirect effects on conflict-affected sex workers’ vulnerability to HIV infection, as it not only predisposes women to exploitation or HIV risk, but may also hinder uptake of HIV preventative and treatments services [16, 38].

#### Police/ criminalization of sex work

In Uganda, Ethiopia, and Sri Lanka, criminalization of sex work led to rushed negotiations with clients due to police presence [11, 29, 31], which significantly increased the odds of client violence [14], further undermining uptake of HIV/STI and SRH services [31]. Among sex workers working in post-conflict Uganda, over one-third (37.3%) reported having to rush negotiations with clients as the result of police presence in the last six months [31], and exposure to incarceration (AOR: 1.93, 95% CI: 1.17–3.20) was found to be positively and independently associated with HIV infection [11]. For refugee sex workers in particular, an arrest for sex work could greatly jeopardize an asylum claim, or result in deportation. As a consequence, the high stakes of getting caught force sex workers to take greater risks with their safety, such as working alone or in secluded areas.

"When I entered the circle of refugees who sell sex, the first thing I learnt was to avoid places the police patrol. When a Ugandan prostitute is arrested, the police have sex with her if she wants to be released without trying. What will happen to me, a refugee?" (31-year-old sex worker from Congo) [23].

Criminalization further propagates an environment in which violence against sex workers is tolerated, resulting in severe vulnerabilities in which sex workers are unable to seek protection from law enforcement authorities [11, 23, 29, 31, 32]. In Uganda, many sex workers revealed that they failed to report human rights violations to police, for fear of re-victimization from public disclosure of their sex work [23]. Numerous instances were documented in which perpetrators of ‘hate-crimes’ against sex workers went unpunished because the victim refused to report the case. In this way, current legal responses to sex work may inadvertently protect perpetrators of violence, and simultaneously expose sex workers to injustice by curtailing access to mechanisms of legal redress [23].

Displaced persons in Uganda were found to have varying levels of awareness of the legal frameworks governing sexual conduct and relationships within their contexts of displacement [23]. In post-conflict states, criminalization has been revealed to undermine effective HIV and harm reduction programmes, by driving key populations underground, and creating environments in which abuses against sex workers’ human rights are tolerated [51, 52]. Further, incarceration may elevate the risk of HIV transmission through a lack of access to condoms, harm reduction supplies, or antiretroviral medicines, as well as through increased vulnerability to human rights violations or sexual assault during detention [52].

#### Access and uptake of HIV/STI and SRH services

During acute stages of conflict when food insecurity, sanitation, and related infectious diseases (e.g., cholera) remain pressing priorities, provision of HIV/STI and SRH services may be negatively impacted, both for the general population and for key populations, including sex workers. Unfortunately, our review identified very few studies describing the nature of HIV/STI or SRH services offered or utilized by sex workers within conflict or post-conflict settings. Available evidence generally indicated that conflict-affected populations face unique obstacles to accessing HIV/STI information, treatment, condoms, and other SRH services. For example, migrant populations often face substantial barriers to access, including the potential for being turned away by providers on account of their status as foreigners [26]. In addition to these already pervasive barriers, evidence suggests that access to HIV and SRH services in conflict-affected settings may be particularly challenging for sex workers, due to the additional marginalization, discrimination, and stigma sex workers face on the basis of their occupation [3]. In post-conflict Gulu, northern Uganda, and over half (55.5%) of sex workers reported experiencing difficulty accessing condoms [11]. In general, HIV, STI and SRH services for conflict-affected sex workers were found to be extremely limited, or where available, lacking in scope, scale-up, and reach (e.g., local-level condom distribution, occasional offers of HIV testing) [3, 11].

##### HIV/STI prevention

Limited exposure to HIV/STI prevention information, condom promotion, and low general HIV knowledge among conflict-affected sex workers was found across multiple settings, including Somalia, Uganda, and Afghanistan [31, 35, 48, 53]. In an epidemiological study of 218 female sex workers in Hargeisa, Somalia, 93% lacked accurate knowledge on HIV/STI prevention, and 28% had never used a male condom [48]. In a study seeking to asses HIV awareness, knowledge, and condom use among women sex workers in Jalalabad, Kabul, and Mazar-i-Sharif, Afghanistan, fewer than 60% of the 520 sex workers interviewed had heard of condoms [53]. Of those who had, only half had ever used a male condom [53]. In a study of 400 young women sex workers in post-conflict Gulu, Uganda, 59.25% reported difficulty accessing condoms or contraceptives, and approximately one-third had never received a condom demonstration [31]. A study conducted by the same group in Gulu Uganda, found that 83.3% of post-conflict sex workers reported inconsistent condom use in the prior six months, for both one-time and repeat clients [11].

One study investigating patterns of condom use with paying sex partners among refugees/IDPs indicated the potential for increased uptake of HIV/STI prevention services during the acute stage of conflict, compared to the post-conflict rehabilitation stage [49]. In a study comparing refugees in the Lugufu refugee/IDP camp at Tanzania’s western border with the DRC to their surrounding host villages, condom use with paying sex partners was quite low (44%) among 15-24 yr. olds in the camp, but was substantially higher than rates in the surrounding villages (25%) [49]. As refugee/IDP camps are heavily influenced by UN and international aid organizations, the authors posited this may be due to an influx of external aid and health services (e.g., condoms, sexual health information) in some camps [49]. However, we did not identify any studies specifically comparing condom use or other indicators of HIV/STI prevention access among sex workers themselves across various stages of a conflict (i.e., acute, post-conflict).

##### HIV/STI testing and treatment

Despite high rates of STIs (17.0%) among sex workers in post-conflict northern Ethiopia, STI treatment was quite low with only 9.6% of those with history of an STI reporting seeking treatment [32]. In Somalia, the majority of sex workers had never been tested for HIV and were unaware of their status. Low awareness of testing locations and concerns regarding the confidentiality of testing were identified as primary barriers [35, 54]. In Gulu, northern Uganda, 92% of women sex workers reported having tested for HIV in the past 6 months, while only 43% reported testing for STIs. This aligns closely with the 40.25% reporting history of an STI infection in the past 6 months, indicating that while testing for HIV may be practiced preventatively, STI testing appears to be done more symptomatically [31]. Of note, this study also found that HIV testing was more common among women who used dual contraceptives than women who had not [31].

##### SRH services

Despite the likely challenges and unique barriers faced by sex workers seeking to access SRH services within conflict-affected settings, little evidence was identified pertaining to this. Only two studies of sex workers’ access and use of other SRH services such as contraception or pregnancy terminations were identified amongst conflict-affected sex workers. An epidemiological study of hormonal contraception use (i.e. birth control pills, Depo-Provera injectables, or implants) among 400 conflict-affected sex workers in Gulu, northern Uganda, found that less than half (49.8%) reported ever using these contraceptives, 10.3% had never used condoms for pregnancy prevention, and 45% reported having ever used dual contraceptives (e.g., birth control pills and condoms at the same time) [31]. In northern Ethiopia, 27% of sex workers reported a history of at least one elective abortion, with 35.3% of these women reporting more than one pregnancy termination [32]. In this same study, 69.0% of sex workers acknowledged history of any type of contraceptive use [32].

#### Policy and programmatic responses to HIV and SRH in sex work in conflict-affected settings

Sex work specific recommendations are largely absent from international guidelines regarding HIV/STIs or SRH in conflict and emergency settings, including those of the United Nations High Commissioner for Refugees (UNHCR), The Joint United Nations Programme on HIV and AIDS (UNAIDS), United Nations Security Council, and the World Health Organization (WHO). Currently, United Nations agencies (such as UNHCR and UNFPA), bilateral donors, and non-governmental organizations offer policy and programme support for the provision of HIV/STI and SRH services to refugees and IDPs in emergency settings. The Inter-Agency Working Group (IAWG) on Reproductive Health in Crisis, spearheaded by UNHCR and UNFPA, has produced a manual specific to humanitarian settings that serves as a guide to SRH and HIV/STI services beginning with the onset of an emergency, and continuing as the situation stabilizes [55]. *The Inter-agency Field Manual on Reproductive Health in Humanitarian Settings* incorporates technical standards set by the WHO, and advocates for increased HIV/STI and SRH services for displaced populations as part of broader primary health care activities. It has identified programmatic strategies to facilitate this process, including implementation of the *Minimum Initial Service Package (MISP),* a set of priority actions in response to the life-saving reproductive health needs of populations at the onset of an emergency. Services included in the MISP for emergency situations include: supplies for infection control, safe deliveries and management of obstetric emergencies, treatment for victims of sexual violence, condoms, oral and injectable contraceptives, drugs for the treatment of STIs, emergency contraception and HIV post-exposure prophylaxis for survivors of rape, and manual vacuum aspiration equipment for the treatment of post-abortion complications [56]. The manual additionally includes guidance on strategies and priorities that should follow when the situation has stabilizes, including adolescent reproductive health, family planning, maternal and newborn health, comprehensive abortion care, gender-based violence, management of sexually transmitted infections, and HIV. While the need for tailored programming or policy recommendations specific to key populations, such as sex workers are largely absent, a number of chapters (e.g. family planning, STI, HIV) do provide insight on how to address the needs of sex workers most effectively. Examples include suggestions to integrate violence reduction strategies in sex work settings, to consult with local staff about how condoms can be made available to sex workers in a culturally sensitive way, to offer regular STI screening services, to work with law enforcement to ensure sex workers have the ability to protect themselves and to ensure safer sex practices by their clients, and to include them from the start in programme design, implementation and monitoring [55]. An overwhelming challenge to provision of these services which is also elucidated, is the reality that health centres in countries with laws against prostitution or discriminatory practices against people engaged in sex work (i.e., the majority of countries worldwide), do not offer services to sex workers [55].

The *Prevention and Treatment of HIV and other Sexually Transmitted Infections for Sex Workers in Low- and Middle-income Countries,* the product of a joint consultation between the WHO, UNFPA, UNAIDS, and the Global Network of Sex Worker Projects (NSWP), summarizes best practices for evidence-based HIV/STI programming for the general population of sex workers [57]. Research and consultation with sex work groups and key humanitarian and global health organizations could be useful to assess the extent to which key principles and practices described in this document (e.g., voluntary HIV testing, decriminalization, community engagement) may be applicable to conflict-affected settings, as well as to articulate and develop strategies to address the unique challenges faced by sex workers in these settings.

Initial steps have been taken to more explicitly address HIV/STI and SRH within the context of sex work in some refugee programmes in the East and Horn of Africa, Latin America and parts of Asia [3]. Based on these experiences, and under the overall framework of the *UNAIDS Guidance Note on HIV and Sex Work*, UNHCR and UNFPA produced a brief *Technical Note on HIV and Sex Work in Humanitarian Settings* [3]. Its primary objective is to inform humanitarian agencies of steps that can be taken to integrate sex worker-inclusive programming into emergency responses. Its recommendations reflect experience from a number of different settings, and are meant to be adaptable to many different conditions and cultural contexts. The *Technical Note* highlights three pillars which aim to: [1] assure sex workers’ universal access to prevention, treatment, care and support; [2] strengthen partnerships with sex workers through community consultation; and [3] reduce vulnerability and address structural issues. The *Technical Note* also addresses “key activities per phase,” which assign importance of activities for the preparedness, emergency, and stabilized phases of a conflict or humanitarian emergency [3]. While these recommendations offer valuable guidance for staff in the field, it is largely up to programme staff to adopt these strategies, and further development of policy guidance to explicitly protect the distinct needs and rights of sex workers in conflict settings may be needed.

Strong global evidence indicates that community empowerment approaches to HIV programming for sex workers, including specific efforts to carefully involve consultation, leadership, and partnership with sex workers in the design and provision of services, are linked to improvements in service uptake and access [3, 58]. A growing number of countries that have scaled up sex worker-led/inclusive interventions have reported stabilization, and even reversal, of their HIV epidemics [3]. Despite the demonstrated effectiveness of community-based sex work HIV prevention programmes, few countries have scaled-up such initiatives [59]. In low and middle-income countries, HIV programmes have traditionally followed generalized approaches, with insufficient attention being paid to the individuals and groups at highest risk of acquiring and transmitting HIV/STIs.

In light of evidence that general-population local health services may not be equipped to meet sex workers’ needs and circumstances [23], there remains a need for targeted approaches to offering and delivering HIV, STI and SRH services for sex workers in displacement and post-conflict contexts, with a particular need for programming efforts that acknowledge and address the diversity of conflict-affected sex workers, including both cis-gender women and those who identify as gender/sexual minorities.

## Discussion

This review highlighted a paucity of sex worker-focused research and interventions in conflict and post-conflict settings. Most of the studies identified were from post-conflict sub-Saharan Africa. Despite our best efforts to identify qualitative or quantitative studies elucidating the specific challenges to health access faced by sex workers during the acute stage of conflict, such studies were largely absent from the peer-reviewed literature, likely due to the substantial practical and ethical challenges involved with collecting such information during complex emergencies. However, available evidence indicated that within conflict-affected settings, sex workers’ capacity to engage in HIV/STI prevention and access SRH services can be undermined by social and structural determinants including widespread violence and human rights violations, the collapse of livelihoods and traditional social structures, high levels of displacement, and difficulties accessing already scant health services due to stigma, discrimination and criminalization.

Unprotected sex between male clients and female sex workers was posited as the primary means of HIV acquisition and transmission in a number of conflict-affected countries under study [30, 35, 48]; yet few, if any studies, examined the HIV burden or social and structural conditions that shaped HIV vulnerability or reduced access to SRH services.

Evidence highlighted the association of conflict-related mobility and displacement with increased engagement in sex work, yet failed to explicitly identify the links between displacement/migration and HIV/STI risk or access to care among this group. While some women intentionally migrate for sex work, others engage in sex work to meet subsistence needs during conflict or migration [60]. Given that migration and displacement are associated with increased socio-economic impacts, social isolation, gender inequalities, and stigma and discrimination [60], HIV/STI risks and barriers in access to care are likely to be amplified among vulnerable groups (like sex workers) in conflict and post-conflict environments. For example, social isolation may pose barriers to the development of support networks to mitigate risk among mobile populations. Evidence from Mexico and Central America has suggested that among sex workers, the formation of community networks - an important pillar of HIV prevention in other contexts - is hindered by the constant mobility of sex workers, who “cannot establish trusting relationships with each other or with others […] [and consequently] cannot demand protected and secure working conditions” [61]. Further research is required which assesses the unique ways in which contexts of mobility and displacement impact HIV/STI risks for sex workers in conflict and post-conflict environments.

Sex work is criminalised in 116 countries globally, with 27 countries in sub-Saharan Africa having official legislation criminalising sex work and ‘prostitution’ [62]. The structures of social stigmatization which criminalisation reinforce inhibit sex workers’ ability to protect themselves from violence and health risks including HIV and other STIs [63]. The illegality of sex work has additionally been understood to lend legitimacy to ongoing abuse and humiliation of sex workers [64]. Numerous qualitative studies reveal the alarming scope and nature of human rights abuses committed against sex workers by local military and police officials themselves:

“There was this time when I was arrested by six policemen. They afterwards demanded sex from me. One of them threatened to stab me if I refused. I ended up having sex with all of them and the experience was so painful.” (26 yr. old male sex worker, Mombasa, Kenya) [51].

“They were policemen. There’s a car park next to the flat and they took me there and they took turns.” (26 year old female, Bulawayo, Zimbabwe) [51].

This continues to be a major challenge in many post-conflict settings where key populations (e.g., gender/sexual minorities such as MSM) are harshly criminalized. For example, numerous sub-Saharan African nations (e.g., Uganda, Kenya, Zimbabwe) have a history of impeding sex workers human rights (e.g., the right to assembly) [14] and have imposed increasingly harsh and draconian criminal laws targeting key populations. In many cases, the political persecution and stigmatization of key populations can foster the perception that antiretroviral therapy (ART) should not be ‘wasted’ on sex workers, a group most in need of treatment [14, 64, 65]. In states where sex work is policed, individuals may fail to access available services for fear of disclosing their so-called ‘non-conforming sexual practices’. A qualitative study of gender/sexual minority refugees engaged in sex work in Uganda highlighted that often alternative sexual cultures (including sex workers, transgender individuals, and men who have sex with men), are silenced and marginalized within current structures of programming, policies and service delivery [23].

In additional studies, sex workers in non-conflict areas have noted the influence broader contexts of criminalization, marginalization, and social exclusion have had on their experiences interacting with the healthcare system.

“We are despised in the hospitals. They [providers] say, ‘We don’t have time for prostitutes’ and they also say that if one prostitute dies then the number reduces.” (27 yr. old female sex worker, Kampala, Uganda) [64].

“When I fell sick and went to a health centre and they realised that I was a sex worker, they did not treat me like a human being. When the health worker came to attend to me […] I was told that he had no time for me. So I left without getting treatment.” (19 yr. old female sex worker, Mombasa, Kenya) [64].

“We were in the queue with everyone else when suddenly one of the nurses came out and loudly said ‘the sex workers who have come … please go and queue at the back of this line, we will attend to you last” (29 yr. old female sex worker, Zimbabwe) [65].

We found little information regarding the lived experiences of conflict-affected sex workers as they attempt to access SRH and HIV services in these challenging environments, and about how commonly such access is compromised or even denied outright, information that is greatly needed to optimise the design of services for this at-risk population [64]. Given the widespread acknowledgment of barriers in access to SRH and HIV services for sex workers in non-conflict environments, paired with the pronounced vulnerability conflict and mobility impart on social isolation and stigmatization of this group, this presents a considerable gap within the literature.

Further research focused on social and structural factors such as migration and displacement, sex work environments, economic contexts, political and legal inequities, and human rights abuses suffered by sex workers in conflict-affected settings is recommended. Rigorous studies investigating the specific links between conflict exposure, these socio-structural forces, and health outcomes among conflict-affected women at risk of, or living with HIV, including sex workers, remain urgently needed. Research detailing HIV/STI or SRH services available during conflict, and the challenges, facilitators, or inequities sex workers face in their interactions with these programmes and services is sorely needed. Given the paucity of studies explicitly articulating specific conflict experiences or their links to HIV/STI risks and programme access among sex workers, further research focused on better understanding the specific impacts of conflict experiences (e.g., war-related human rights violations) are recommended. We see this review as a preliminary step in better understanding these issues, and recommend future research and policy work be focused on evidence-based interventions and the impact of programmatic changes.

Findings from this review suggest an urgent need to scale up access to quality, accessible, and non-stigmatizing SRH and HIV programmes for sex workers in conflict-affected areas. Considerations of the legal and policy environments in which sex workers operate, and actions to address the important role of stigma, discrimination, social isolation, and violence targeting sex workers is needed. Criminalized work environments continue to undermine HIV prevention strategies, and pose serious barriers to utilization of SRH services among sex workers globally [9, 11, 14, 23, 31, 66]. Policy shifts away from criminalization, towards a human rights-based model for sex workers, remain critically needed, during armed conflict and in the fragile peace that follows. As significant barriers in access to care appear to be for sex workers in post-conflict settings, local level multilevel strategies which address factors at different levels, including individual risk factors, partner-level, social determinant, etc., may be most successful to address the severe structural barriers impeding sex workers’ health and human rights in these settings.

Future public health interventions in conflict and post-conflict settings should seek to promote peer-led programmes and initiatives on the ground, in addition to recognizing sex workers as key stakeholders/experts in high-level policy consultations. In Kenya and Uganda, UNHCR and implementing partners have worked closely with sex workers in the development of programmes, based on sustainable and improved comprehensive services including HIV and reproductive health, community social services and livelihood interventions [67]. Both cases provided evidence that much can be achieved within a six-month period: sex worker-led organizations and community outreach can be established, confidential and respectful healthcare services can be provided, and protection systems strengthened. These examples illustrate how the active engagement and involvement of sex workers is not only possible, but can also lead to improved quality of HIV prevention measures [3]. Future public health interventions should additionally seek to target other vulnerable groups, including refugees and migrants broadly, men who have sex with men, transgender individuals, and the partners and clients of sex workers (e.g. military, peacekeepers, and police) in conflict and post-conflict environments. In Cambodia, for example, a brand of condoms marketed specifically to the military since 1997 has helped reduce rates of unprotected sex between Cambodian soldiers and sex workers, from 70% to 54% [33]. Clients have an important supportive role to play in supporting sex worker programmes, which should not be understated. If left out, they may undermine efforts to reduce safety concerns, sexual exploitation, and gender based violence. Community-led activities should be strengthened within the community which promote responsibility not only among sex workers, but also their partners [67]. Lastly, while the importance of better linking HIV and SRH services in the general population and for sex workers has been previously discussed, demonstrated to be feasible, and linked to improved health outcomes in a number of settings (e.g., linked to increased condom use, HIV testing, and lowering HIV and STI rates) [45, 68], special efforts to develop and implement ‘best practices’ for such service integration within the unique challenges faced by conflict-affected settings are required.

### Limitations and directions for future research

Since too few studies exist in this area to have employed a systematic review or meta-analysis, we employed a comprehensive review methodology to meet our objectives. Challenges arose in distinguishing the fluid boundaries between acute conflict and post-conflict states, and conflations of sex work with transactional sex (i.e., exchange of non-monetary commodities with non-commercial partners) or human trafficking for the purposes of sexual exploitation. While many studies defined transactional sex as the exchange of sex for both monetary and non-monetary commodities, when they focused specifically on non-monetary exchanges with non-commercial partners they were excluded from this review. Most studies did not explicitly articulate the ways in which working and living in conflict and post/conflict settings may impact risk of HIV/STIs and access to SRH services for sex workers, creating large gaps in our understanding of distinct pathways in these contexts. Given that very few studies employed longitudinal designs, studies incorporating temporal aspects that would more easily allow inferences regarding the impacts of conflict on sex workers’ health and access to care, such as studies that compare pre and post-conflict conditions and health outcomes, may be particularly useful. In addition, many people who exchange sex to supplement their incomes in humanitarian and post-conflict settings do not self-identify as sex workers, making research and surveillance in this area a challenge. To the best of our knowledge, this review is the first synthesis of evidence addressing social and structural drivers of HIV/STIs and SRH among conflict-affected sex workers.

## Conclusions

Results from this review demonstrate the presence of numerous social and structural factors increasing HIV/STI risks, and creating severe barriers in access to HIV and SRH services for conflict-affected sex workers. Significant gaps in context specific sex worker-focused research, policy, and programming were identified. While recommendations are available to guide interventions with sex workers in conflict and post-conflict settings, universal, national, regional, and local policies and regulations are largely absent, and the degree to which sex workers are accessing SRH services during emergencies remains unclear. A paradigm shift from the behavioural and biomedical approach to HIV among sex workers, to a health and human rights approach, in displacement and post-conflict settings is strongly recommended.

## Abbreviations

ART: Antiretroviral therapy
GBV: Gender based violence
HIV: Human immunodeficiency virus
HIV/AIDS: Human immunodeficiency virus / acquired immunodeficiency deficiency syndrome
IAWG: Inter-Agency Working Group (IAWG) on Reproductive Health in Crisis
IDP: Internally displaced person/people
MISP: Minimum initial service package
NSWP: Global Network of Sex Worker Projects
PMCTC: Prevention of mother-to-child transmission
SRH: Sexual and reproductive health
STIs: Sexually transmitted infections
SW: Sex worker
UNAIDS: Joint United Nations Programme on HIV/AIDS
UNFPA: United Nations Population Fund
UNHCR: UN Refugee Agency
VCT: Voluntary counselling and testing
WHO: World Health Organization
